# Preservation of Organs to Be Transplanted: An Essential Step in the Transplant Process

**DOI:** 10.3390/ijms23094989

**Published:** 2022-04-30

**Authors:** Maryne Lepoittevin, Sébastien Giraud, Thomas Kerforne, Benoit Barrou, Lionel Badet, Petru Bucur, Ephrem Salamé, Claire Goumard, Eric Savier, Julien Branchereau, Pascal Battistella, Olaf Mercier, Sacha Mussot, Thierry Hauet, Raphael Thuillier

**Affiliations:** 1 Biochemistry Department, CHU Poitiers, 86021 Poitiers, France; maryne.lepoittevin@univ-poitiers.fr (M.L.); sebastien.giraud@chu-poitiers.fr (S.G.); raphael.thuillier@univ-poitiers.fr (R.T.); 2 Faculty of Medicine and Pharmacy, University of Poitiers, 86073 Poitiers, France; thomas.kerforne@chu-poitiers.fr; 3 INSERM U1313, IRMETIST, 86021 Poitiers, France; benoit.barrou@aphp.fr (B.B.); lionel.badet@chu-lyon.fr (L.B.); 4 Cardio-Thoracic and Vascular Surgery Intensive Care Unit, Coordination of P.M.O., CHU Poitiers, 86021 Poitiers, France; 5 Sorbonne Université Campus Pierre et Marie Curie, Faculté de Médecine, 75005 Paris, France; 6 Service Médico-Chirurgical de Transplantation Rénale, APHP, Hôpital Pitié-Salpêtrière, 75013 Paris, France; 7 Société Francophone de Transplantation et de l’Ecole Francophone pour le Prélèvement Multi-Organes, 75013 Paris, France; p.bucur@chu-tours.fr (P.B.); e.salame@chu-tours.fr (E.S.); claire.gourmand@aphp.fr (C.G.); eric.savier@aphp.fr (E.S.); julien.branchereau@chu-nantes.fr (J.B.); pascal.battistella@chu-montpellier.fr (P.B.); olaf.mercier@aphp.fr (O.M.); s.mussot@ghpsj.fr (S.M.); 8 Faculté de Médecine, Campus Lyon Santé Est, Université Claude Bernard, 69622 Lyon, France; 9 Service d’Urologie et Transplantation, Hospices Civils de Lyon, Hôpital Edouard-Herriot, 69003 Lyon, France; 10 Service de Chirurgie Digestive et Endocrinienne, Transplantation Hépatique, CHU de Tours, 37170 Chambray les Tours, France; 11 Groupement d’Imagerie Médicale, CHU de Tours, 37000 Tours, France; 12 University Hospital Federation SUPORT Tours Poitiers Limoges, 86021 Poitiers, France; 13 Service de Chirurgie Digestive, Hépato-Bilio-Pancréatique et Transplantation Hépatique, APHP, Hôpital Pitié-Salpêtrière, 75013 Paris, France; 14 Service d’Urologie et de Transplantation, CHU de Nantes, 44000 Nantes, France; 15 Service de Cardiologie et Maladies Vasculaires, CHU de Montpellier, CEDEX 5, 34295 Montpellier, France; 16 Service de Chirurgie Thoracique et Cardio-Vasculaire, Centre Chirurgical Marie LANNELONGUE, 92350 Le Plessis Robinson, France

**Keywords:** kidney transplantation, organ preservation, temperature, oxygen

## Abstract

Organ transplantation remains the treatment of last resort in case of failure of a vital organ (lung, liver, heart, intestine) or non-vital organ (essentially the kidney and pancreas) for which supplementary treatments exist. It remains the best alternative both in terms of quality-of-life and life expectancy for patients and of public health expenditure. Unfortunately, organ shortage remains a widespread issue, as on average only about 25% of patients waiting for an organ are transplanted each year. This situation has led to the consideration of recent donor populations (deceased by brain death with extended criteria or deceased after circulatory arrest). These organs are sensitive to the conditions of conservation during the ischemia phase, which have an impact on the graft’s short- and long-term fate. This evolution necessitates a more adapted management of organ donation and the optimization of preservation conditions. In this general review, the different aspects of preservation will be considered. Initially done by hypothermia with the help of specific solutions, preservation is evolving with oxygenated perfusion, in hypothermia or normothermia, aiming at maintaining tissue metabolism. Preservation time is also becoming a unique evaluation window to predict organ quality, allowing repair and/or optimization of recipient choice.

## 1. Introduction

**Figure 1:**
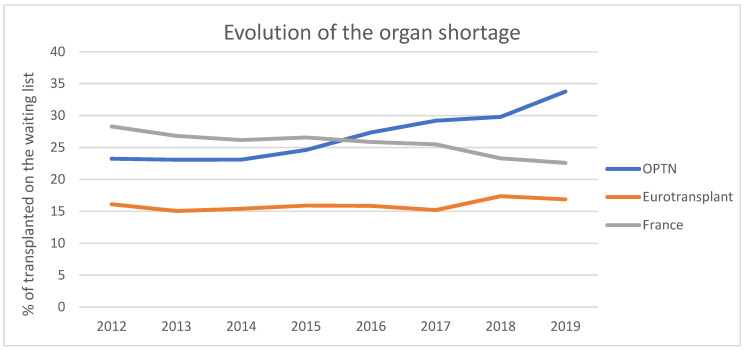
Evolution of the number of organ transplants performed as a percentage of the waiting list in several transplantation areas: France, Eurotransplant and Organ Procurement and Transplantation Network (OPTN).

Transplantation remains the best therapeutic alternative in the event of terminal organ failure, both in terms of quality-of-life and life expectancy, as well as in terms of public health expenditure. The number of candidates for transplantation is constantly increasing and there is now a very important organ shortage (only 15 to 35% of patients on the waiting list receive an organ, Figure 1), particularly due to population ageing and the increasing incidence of cardiovascular diseases, both of which lead to organ failure. 

There are three types of organ donation: (i) living donors (kidney and, exceptionally, part of the liver); (ii) brain-dead donors (DBDs) (kidney, liver, heart, lung, pancreas and intestine); and (iii) deceased donors after circulatory arrest (DCDs) (kidney, liver, lung and heart). In recent years, the contraindications to donation have been revised in order to better meet the demand for organs. Indeed, in order to increase the number of organs available for transplantation, there has been a renewed interest in donation from DCDs [1], which is considered an effective way to increase the number of potential donors [2]. In addition, organ selection criteria for DBDs have been expanded (e.g., for kidney: donor age > 60 years or donor age > 50 years in cases of death from vascular causes, creatinine level > 1.5 mg/dL (137 mmol/L) and/or history of hypertension [3]. Unfortunately, these “new” grafts are more susceptible to ischemia-reperfusion (IR) injury (IRI). IR starts as soon as the organ is no longer supplied by the bloodstream (ischemia), and is enhanced when the organ is revascularized (reperfusion) and can induce a wide array of injury mechanisms which have detrimental effects on both short- and long-term outcome. It is therefore necessary to adapt the means of graft preservation, which is why organ transplantation is in a phase of profound changes. 

The main purpose of organ preservation is to maintain the viability of individual organs ex vivo for a period of time that is commensurate with the type of organ being considered. It must be sufficient for the eventual transport of the organs from the procurement center to the transplant center(s) in order to be allocated according to the degree of priority and/or best tissue compatibility. The management of these organs is very different depending on whether it is a vital organ such as the heart, liver or lung, or the kidney and pancreas for which a delay in the recovery of the graft function is “tolerable” thanks to the availability of substitution therapeutics (dialysis and/or insulin). Preservation also aims at reducing IRI. Importantly, tolerance to IRI varies according to the type of organ and the type of donor.

## 2. The Different Types of Donors

Organ transplant teams determines three categories of donors: living donors, donation after brain death donors (DBDs) and donation after circulatory death donors (DCDs).

The first category is the living donor (kidney and liver). These transplants are not without morbidity and mortality, but their evolution is very favorable and superior in terms of short- and long-term outcomes for the graft and the patient [4]. The development of kidney transplantation from living donors is ongoing, but will likely not meet the increase in demand for grafts, and regarding the liver, it is mostly exceptional and indicated for paediatric patients [5]. 

Because the number of standard criteria donors (SCDs) is not sufficient to meet the demand, it was necessary to resort to alternative sources of organs, such as expanded criteria donor (ECDs) [6,7]. Thus, for the kidney, ECDs are donors either >60 years old or aged 50 to 59 years with at least two of the following three comorbidity criteria: (i) cerebrovascular accident as cause of death; (ii) serum creatinine level > 1.5 mg/dL (137 mmol/L); and (iii) preexisting history of systemic hypertension (Table 1). Extended criteria have also been defined for other organs [7].

DCDs are categorized according to the Maastricht classification (Table 1). Their hearts stop before organs are collected. Kidney, liver, pancreas, lung and, more recently, heart donations are eligible from DCDs. In the case of uncontrolled donation, only kidneys, livers and lungs have been successfully transplanted [2].

**Figure 2:**
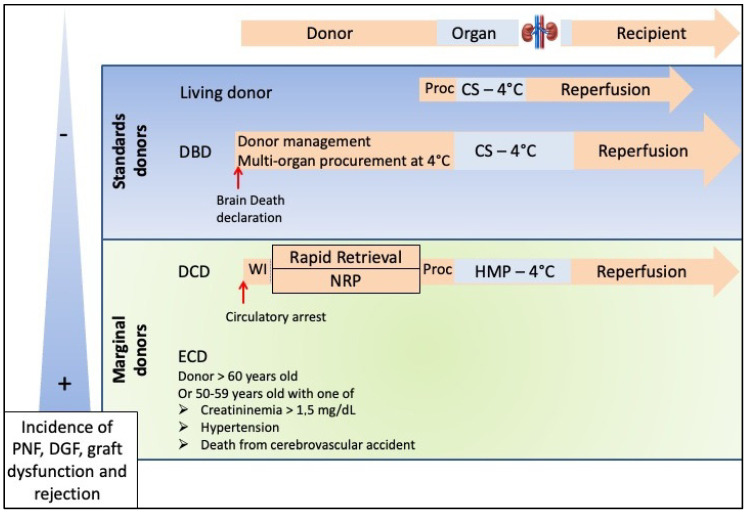
Graft course according to donor type (e.g., kidney). Abbreviations: extended criteria donors (ECDs); deceased donors after circulatory arrest (DCDs); brain-dead donors (DBDs); delayed graft function (DGF); primary non-function (PNF); static conservation at 4 °C (SC); normothermic regional perfusion (NRP); warm ischemia (WI); hypothermic conservation on perfusion machine (HMP). Figure adapted from Franzin et al. [12].

**Figure 3:**
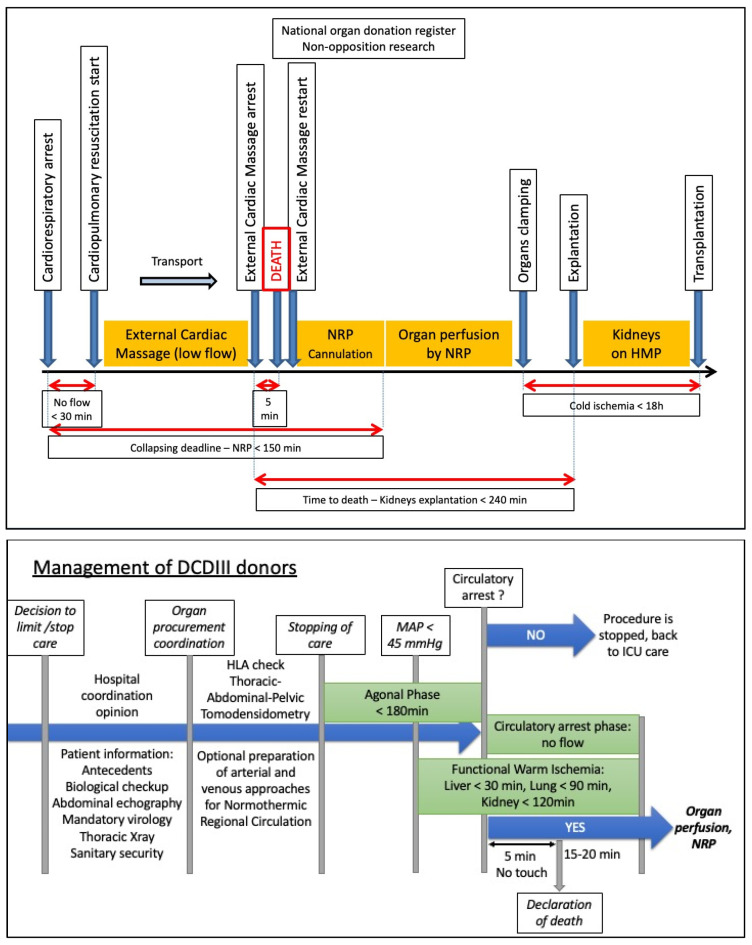
Management protocols for DCDs. **Top:** organization and timeframes for organ retrieval using NRP from donors who have died after circulatory arrest (NRP—normothermic regional perfusion; HMP—hypothermic machine perfusion; MAP—mean arterial pressure). Figure from the French Association of Urology [13]. **Bottom:** management of Maastricht III donors. NRP—normothermic regional perfusion. Figure adapted from the Agence de Biomedecine.

The practice of solid organ transplantation from DCDs poses certain ethical, and pathophysiological problems in relation to cardiac arrest [7]. It is a process involving the donor, organ preservation and the recipient. The management of the donor also involves organizational and ethical aspects. Both the use of the ‘rapid retrieval’ [8] and Normothermic Regional Perfusion (NRP) are possible, with the latter being recommended by consensus in many countries [9], followed by a judicious choice of preservation means and in particular the use of perfusion machines, whose protocols are being optimized in terms of perfusion medium, oxygenation and temperature [7,10]. Our own work demonstrated the importance of timing during NRP, showing that there was a minimal time required to obtain the most optimal organ rejuvenation conditions (4 h) and that either shortening or prolonging NRP could have detrimental effects on graft quality [11]. The role of preservation in the graft journey according to the type of donor (e.g., kidney) is shown in Figure 2. Management protocols for DCD donors are presented in Figure 3.

## 3. Ischemia-Reperfusion

**Figure 4:**
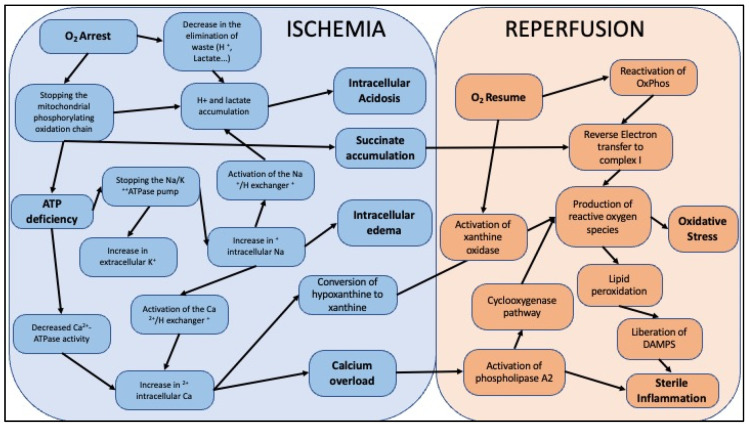
Cellular impacts of ischemia reperfusion. The sequence of events taking place during ischemia reperfusion is displayed, from the arrest of oxygen supply and consequences during ischemia, to the oxidative stress resulting from resumption of oxygen supply and the resulting sterile inflammation.

The use of lower-quality organs, including those from DCDs, has become an established routine but increases the risk of graft dysfunction. This risk is further compounded by IRI, which is unavoidable during the transplantation process [14,15,16]. Graft injury begins in the DBD donor at the time of brain death, which results in a significant systemic inflammatory response syndrome (SIRS). This is characterized by a massive release of pro-inflammatory cytokines, such as tumor necrosis factor (TNF), interleukin (IL)-1 or IL-6, associated with an endothelial hyperexpression of cell adhesion molecules, such as selectins, and ICAM and VCAM integrins [17]. This response is followed by tissue infiltration by activated inflammatory cells, including macrophages and neutrophils. This first phase is thus at the origin of lesions that will initiate the phenomena responsible for the alteration of the primary function recovery and the chronic dysfunction of the grafts. In the case of DCD donors, the lesions begin at the time of functional warm ischemia following circulatory arrest. This initial phase is followed by graft collection. It is marked by a short period of warm ischemia, linked to the surgical process of graft removal, following the clamping of the celiac aorta. This phase is followed by a phase of cold ischemia, secondary to the cooling of the organ by the preservation solution previously maintained at 4 °C and which will remain so in the case of hypothermic preservation. The ischemic phases are characterized by a decrease in the supply of oxygen and nutrients to the organ, and consequently of energy substrates [16,18]. This lack of supply is responsible for a decrease or even a halt in mitochondrial activity, the organelle responsible for the majority of cellular energy production. The lack of oxygen induces a reduction in ATP synthesis, which is responsible for a shutdown of ATP-dependent ion pumps, disrupting cellular ion homeostasis and promoting a series of harmful metabolic processes and cellular changes. This is associated with an increase in intracellular calcium concentration, responsible for the activation of calcium-dependent enzymes such as phospholipases involved in various processes. Depending on the duration of ischemia, these processes lead to more or less reversible mechanisms such as activation of the endothelium and cell death by necrosis or apoptosis. This phase also sees activation of hypoxia adaptation mechanisms, such as the activation of hypoxia inducible factor 1a (HIF1a) [19,20]. This is a transcription factor sensitive to tissue oxygen partial pressure, stabilized during hypoxia and which has the capacity to stimulate the expression of numerous genes whose proteins are pro-angiogenic or activate glycolysis under anaerobic conditions. During transplantation, reperfusion is defined by the revascularization of the ischemic organ and is at the origin of the reintroduction of energy and oxygen supplies. This sudden oxygenation of the ischemic territory is responsible for a process called oxidative stress. It is defined by the production of partially reduced oxygen species which have the characteristic of being reactive towards all the major classes of biological molecules such as carbohydrates, proteins, DNA, RNA and lipids. This process is therefore responsible for cell damage that can lead to cell death. Moreover, endothelial activation and endothelial damage are major processes induced during reperfusion that favor acute or chronic graft dysfunction. Indeed, activation of the endothelium will be responsible for a pro-aggregating state hindering reperfusion and partly responsible for “no reflow”, which is a partial reperfusion of the ischemic territory. Our team has studied this endothelial dysfunction by showing its link to a rarefaction of microvessels responsible, during reperfusion, for chronic hypoxia which is increased in the long term by the establishment of scar tissue: fibrosis [21,22]. Activation of the endothelium will increase during the initial stages of reperfusion and lead to the recruitment of inflammatory cells, which are mainly represented at this stage by monocytes/macrophages and neutrophils. This innate immunity is notably dependent on damage associated molecular patterns (DAMPS), alarmins capable of activating receptors such as toll-like receptors (TLR) and stimulating the inflammatory process. A summary diagram of the main cellular impacts of ischemia-reperfusion is presented in Figure 4. Adaptive immunity is also activated in a second step by the recruitment of T cells. Our team has also demonstrated the role of innate immunity and IL-33 in the context of IR [23]. These IR lesions are more or less severe depending on the type of donor and the conditions and duration of graft storage. For example, grafts from donors in a precarious hemodynamic state or with comorbidity factors such as a history of hypertension, dyslipidemia, or impaired renal function are particularly susceptible to IR lesions, highlighting already-established lesions [24,25]. Our unit has provided data on the role of dyslipidemias [20]. 

Thus, one of the current challenges for the transplant community is to improve the quality of preservation in order to reduce complications related to IR. The use of cold storage conditions for flushing and static cold storage (CS) has been the standard storage technique for many years. However, new donor demographics, such as expanded criteria donors (ECDs) and DCDs, have led to the development of more diverse preservation techniques [26]. We refer the reader to recent reviews on the pathophysiology of the IR syndrome [14,16,27,28].

## 4. Means of Organ Preservation

Static storage in hypothermia without oxygen supply has been the chosen solution for decades. The purpose of hypothermia (between 4 and 6 °C) was to limit metabolism and oxygen requirements [29]. Many solutions of different compositions are marketed (Table 2) and have shown promising effects in decreasing ischemic injury and improving graft quality and function in preclinical animal studies. Our own work highlighted the superiority of extracellular composition for the preservation solution [30], and in particular demonstrated the advantages of using polyethylene glycol as a colloid in the composition of the solution. Indeed, this compound has very interesting properties with regards to the challenges of organ preservation: it does not present the pro-coagulant inconvenient of hydroxyethyl starch, and furthermore it adsorbs to the plasma membrane and creates an ‘exclusion space’ which prevents fixation of immune cells and therefore decreased post-ischemia reperfusion sterile inflammation. This phenomenon is termed ‘immunocamouflage’ [31].

However, clinical evidence is still lacking [28,32,33]. Indeed, the clinical literature does not allow a clear discrimination between solutions, highlighting a lack of large-scale clinical trials. It is also necessary to take into account the specifications of these solutions aimed at counteracting the mechanisms of ischemic injury: oxidative stress, apoptosis/necrosis, inflammation, cellular/tissue edema, alteration of cellular and mitochondrial integrity [32]. 

Today, given the evolution of donors, hypothermic machine perfusion (HMP) is recommended for DCDs [2]. Our group has provided further cognitive insight into the mechanism of machine perfusion protection, chief among which is the maintenance of NO signaling through eNOS activation by AMPK signaling, which improves kidney quality [34]. This technique has been chosen as the standard preservation method for deceased kidneys in The Netherlands in 2016, and the results of this measure have shown that HMP is associated with a significant reduction of DGF across all donor types [35]; however, the benefits on long term outcome appear to be more linked to donor parameters rather than the use of HMP [36]. This positive effect on early outcome was also found in the liver [37], however in the heart, HMP did not demonstrate superiority to standard cold storage [38]. Hence, there is still room for improvement regarding these means of preservation, for instance through oxygenation, temperature and/or pharmaceutical agents.

### 4.1. Temperature

Hypothermia is currently being reevaluated as it is suspected of having deleterious effects. For instance, ex vivo normothermic perfusion (EVNP) maintains cellular processes at physiological temperature. This technology has been widely tested in the UK, Canada, The Netherlands and the United States, and several studies have demonstrated the advantages of EVNP over hypothermia [26,39,40,41,42]. The few existing clinical studies demonstrate that this EVNP technique is both feasible and safe, notably by the Cambridge group [43,44]. This group is conducting a multicenter randomized controlled phase II clinical trial (ISRCTN15821205), which has finished recruitment and results are expected soon. A recent study started to evaluate possible additional benefits of this technique when complementing regular HMP for suboptimal donor kidneys (NCT04882254) [45].

An international comparative study has shown that DCD donor livers maintained under oxygenated hypothermia perfusion (HOPE) or NRP have post-transplant survival rates comparable to those of standard liver [46]. The use of the normothermia perfusion machine (NMP) on human livers considered acceptable for transplantation has been evaluated in several clinical studies [47]. The results of a European randomized clinical trial of normothermic preservation in liver transplantation showed that peak serum aspartate aminotransferase was lower in the NMP group compared to the static hypothermia group [48]. A recent review highlights the superiority of NMP over HMP in the liver, with additional benefits regarding length of hospital stay and the risk of primary non-function on all donor types [37].

For the lung, several clinical trials (NCT01190059 and NCT01963780) have shown that EVNP is a real alternative for high-risk donor lungs in transplantation, with similar results to those obtained with conventionally selected lungs [49,50]. In addition, normothermic perfusion preservation of hearts from DCDs is a realistic possibility with possibly longer preservation times [51]. Another aspect is the speed of reaching EVNP and its prolonged duration, which seem to be essential factors for organ protection [43,52,53,54,55]. Finally, heart preservation by NMP is feasible and appears to preserve the hearts from extended criteria and DCDs with comparable results to SCS; however, the technique is not as safe and simple as HMP [38].

Another strategy is investigated: controlled oxygen rewarming (COR). This technique was performed in humans, using a kidney graft from an extended criteria donor. The kidney was preserved during ex vivo perfusion by a machine from hypothermia to normothermic conditions (35 °C with 100% oxygen), and the results seem promising [56]. 

Several reviews complement the data on the value of temperature and perfusion machines, especially in marginal donors [7,57,58,59,60].

### 4.2. Oxygen

Several works emphasize the provision of oxygen to improve hypothermic perfusion [61,62]. Several solutions exist, but it should be noted that the addition of oxygen could have double-edged effects, as excessive oxygenation can induce the production of free radical oxygen species [63], although this side effect seems minimal. Preclinical models have shown that the provision of gaseous oxygen during organ perfusion can improve renal graft quality, as observed by (i) a more rapid increase in renal flow, a decrease in lactate and an increase in ATP, (ii) improved creatinine clearance, (ii) improved recovery of organ function, and a decrease in interstitial fibrosis [63,64,65,66,67]. Clinical studies have been initiated, such as the “COMPARE” clinical trial evaluating oxygenated PMH by the Consortium for Organ Preservation in Europe (COPE; COMPARE trial, ISRCTN32967929). Published in November 2020, this study showed that oxygenated hypothermic machine perfusion (HMPO2) of DCD kidneys is safe and reduces post-transplant complications [62], but the estimated glomerular filtration rate (eGFR) at 12 months was not significantly different between the HMPO2 and HMP groups. However, potential beneficial effects of HMPO2 were suggested by the authors with the analysis of secondary outcomes. More nuanced results were obtained with the NCT03378817 clinical trial [68,69]. Regarding the benefits, recent work highlighted that oxygenation is more relevant when used in combination with normothermia [70].

Another alternative is the use of oxygen carriers during organ preservation. A recent review on the use of oxygen carriers has been published [71]. Different molecules (Hemopure, M101, HbV, Erythromer and Hemerythrine) have been studied with interesting results [72,73,74,75]. Our own work with M101, in a pig kidney autotransplantation model, demonstrated superiority of M101-supplemented solutions, lowering the peak of serum creatinine and increasing the speed of function recovery. Furthermore, tissue integrity was better maintained, with less brush border loss and endoluminal cellular detachment. Following the animals for 3-month follow-up period, we showed that M101 supplementation was beneficial in term of survival, function and the progression of interstitial fibrosis and tubular atrophy [76]. 

These preclinical studies led the molecule to be accepted for clinical trials, with the results of the safety trial (OXYOP), published in June 2020, showing that the use of marine worm hemoglobin M101 is safe and has promising efficacy data (no immunological, allergic or prothrombotic effects were reported). The authors reported less delay in recovery of renal graft function, fewer dialysis sessions per patient in the first month, a decrease in creatinine levels over the first 7 days, and a better time to reach creatinine <250 μmol/L in the M101 group compared with the contralateral group without M101 [77]. These results show that oxygenation during preservation could be beneficial, and the combination of the two approaches has been tested for the liver [78,79]. Our own team tested the combined benefits of oxygen and M101 in a pig kidney autotransplantation model with long term follow up, demonstrating a degree of additivity between the two strategies, chiefly regarding chronic development of tissue injury [61].

Interestingly, a group in Guangzhou evaluated a procedure, called ischemia-free organ transplantation (IFOT), to optimize organ transplantation in humans. They demonstrated that the liver and kidney can be collected, preserved and implanted under continuous normothermic oxygen machine perfusion to limit ischemia. Under these conditions, the liver did not suffer in the post-reperfusion period, with minimal injury, little inflammation and no complications [80], and nor did the kidney, with lower markers of injury and rapid functional recovery [81].

A recent review offers an in-depth exploration of the impact of oxygen in organ preservation [82]. Additionally, various perfusion techniques are being developed. In addition to hypothermic perfusion, normothermic and subnormothermic perfusion, controlled oxygen reheating, and more recently supercooling are being developed [58,83,84,85,86]. 

Ex vivo preservation is also a window to evaluate organs and implement therapeutic strategies exerting antioxidant, anti-inflammatory and anti-apoptotic activities, siRNA delivery or even cell therapy. The combination of different perfusion methods is also explored, which implies a tailor-made organ preservation in connection with the evaluation of the organs but also the donor in order to best match the recipient [10,69,86]. In all, this extracorporeal period of organ preservation is a critical time that requires improvements to resuscitate and optimize graft quality. It is a period that can be used to identify diagnostic biomarkers of ischemic damage that predict organ transplantation outcomes. Collectively, these various advances point to the possibility of personalized and predictive medicine and a new era for organ preservation.

### 4.3. Pharmacological Agents

The preservation period is a very interesting therapeutic window allowing the use of targeted pharmacological agents. One of the difficulties is to have agents that can act in static or perfusion conditions and in particular in hypothermia. The development of normothermia and the evolution of perfusion media should allow the use of molecules that can act in a more physiological manner. Several recent reviews have presented the different pharmacological agents that can be used [12,59,87].

Among these, two strategies stand out the most:

Mitonchondrial protection targeting the factor eIF5A (eukaryotic initiation factor 5A) and its enzymatic activation step (hypusination) using a spermidine-like compound (GC7). This molecule induces a drastic fall of oxygen consumption associated with a reduction of the mitochondrial membrane potential and a decreased expression of mitochondrial complexes [88]. We tested this strategy in a porcine preclinical model of kidney transplantation [88], showing that treating the donor with GC7 before renal pedicle clamping greatly improved the recovery of the renal function at least up to 3 months. We further tested the protective properties of this molecule in a brain death model of pig kidney transplantation, again demonstrating the important of preconditioning, through mitonchondrial targeting, to improve success [88,89].

Mineralocorticoid receptor modulation, which can lead to a better management of kidney function, improved protection against inflammation and maintenance of the integrity of the vascular bed, a key target of ischemia reperfusion injury [21,90]. In a preclinical study on a large White Pig, we showed that Soludactone (a Mineralocorticoid receptor antagonist) was highly efficient to prevent ischemia reperfusion injury and acute kidney injury both on the short and long term [91]. 

Numerous other candidates have been studied over the past few decades but with very few clinical outcomes. Moreover, there is a need to target the primary pathophysiological mechanisms involved during preservation without ignoring the donor and recipient.

## 5. Perspectives and Conclusions

**Figure 5:**
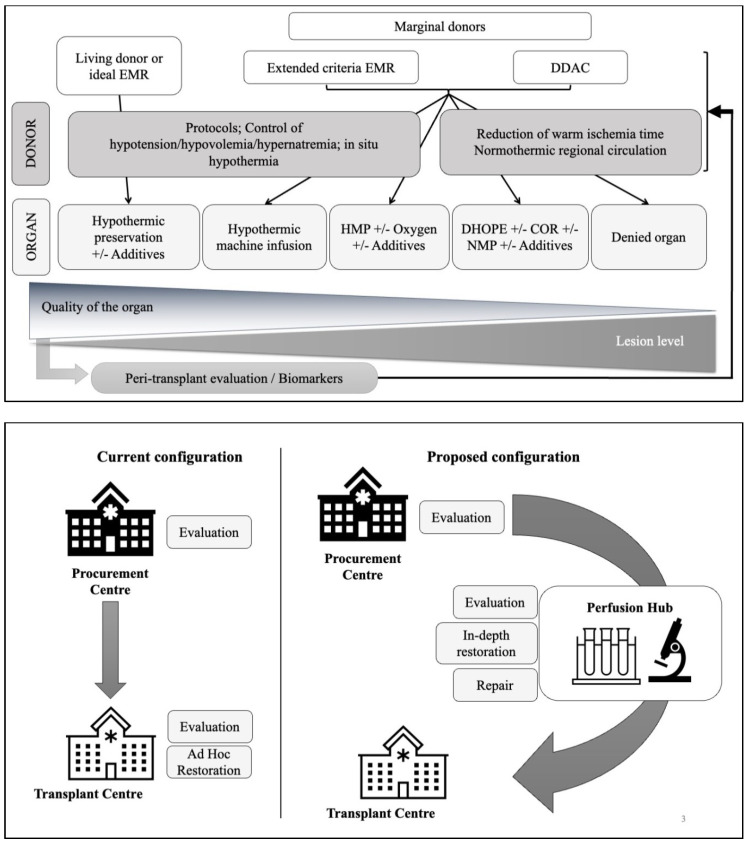
Proposed organ-management algorithms for transplantation. **Top**: Decision-making algorithm based on the quality of the organ to be transplanted. Depending on the quality of the organ, and thus its degree of damage, it could be directed towards adapted management protocols, notably at the level of the donor in the event of characterization of this quality as soon as brain death, but also once the removal has been carried out with a wide range of technical and molecular options. **Bottom**: proposal for the organization of the organ pathway towards perfusion centers, allowing an ad hoc evaluation of the organ and the implementation of high-quality preservation/repair protocols, thanks to the concentration of means.

Given the diversity of organ quality in DCD, and the fact that donor inclusion criteria will need to be expanded to address the growing organ shortage, new quality classification tools are needed. There is an increasing need for pre-transplant prognostic models of recipient outcomes based on accurate surrogates of organ quality, not only for health care organizations responsible for transplant allocation, but also for physicians and patients. This would increase the organ pool of deceased donors, improve donor-recipient matching, and facilitate decision making for living donors (Figure 5, top). 

One such tool is the Kidney Donor Risk Index (KDRI), which takes into account demographic and clinical characteristics related to the donor and transplantation [92,93]. Another such algorithm is the iBox, which has a much better area under the ROC curve (mean c-index: 0.8), but is a post-transplant tool [94].

Other means such as the metabolic, transcriptomic or metabolic approach are interesting perspectives to optimize the organ donation process. A better knowledge of the physiopathology specific to each type of donation, as well as the implementation and application of specific protocols, would eventually allow a personalized management of the grafts from the selection of the donor to their conservation and transplantation.

There is a need for teams involved in this area to consider networking in order to have coordinated protocols. There is also a need to establish international registries and to target key questions in unified international efforts. The metabolic aspect is emerging for both the tissues that make up the organs and the inflammatory cells involved. A recent study indicated that the liver and kidney have a markedly different metabolic profile in deceased organ donors, with a preference for alanine production in the liver and lactate in the kidney [95]. Expression of the renal lactate-transporter gene MCT4 increased as a support for the changing metabolic profile. Recent work on the metabolic reprogramming of innate immune system cells has placed cellular and mitochondrial metabolism at the center of the macrophage differentiation program [95]. Recent studies showing that cells of the innate immune system can undergo functional reprogramming, facilitating a more rapid and enhanced secondary response, support a new concept called innate immune memory or trained immunity [96]. Trained immunity not only involves the reprogramming of intracellular immune signaling in innate immune cells, but also induces profound changes in cellular metabolic pathways such as glycolysis, oxidative phosphorylation, fatty acid and amino acid metabolism [97]. Given the role of innate immunity in IR, these findings open up avenues of interest for research that could explain, for example, the link between acute injury and the development of chronic injury. 

The final point to emphasize, given the complexity of organ assessment and management, with the multiplicity of investigative tools and preservation techniques, would be the creation of Organ Hubs (Figure 5, bottom), strategically located within an organ donation/transplantation network. This would be a one-stop shop that would allow a greater concentration of resources to obtain the tools and therapeutics necessary to provide the most up-to-date management protocols and thus increase transplant success.

## Figures and Tables

**Table 1:** Criteria for deceased kidney donors: SCDs, ECDs and DCDs.

Categories	Short Description	Description
Standard-Criteria Donor (SCD)	Donor under 60 years of age and do not meet any of the criteria of Expanded Criteria Donors (ECD)	
Expanded Criteria Donor (ECD)	Donor either > 60 years or aged 50 to 59 years with at least 2 of the following three criteria: (i) cerebrovascular accident as cause of death; (ii) serum creatinine level > 1.5 mg/dL (137 mmol/L); (iii) preexisting history of systemic hypertension	
Uncontrolled DCD I	Found dead I a. Out of hospital I b. In the hospital	Unexpected circulatory arrest with no resuscitation. Can donate tissue (cannot donate organs).
Uncontrolled DCD II	Cardiac arrest in front of a witness II a. Out-of-hospital II b. In-hospital	Unexpected circulatory arrest with failed resuscitation
Controlled DCD III	Withdrawal of life-sustaining therapy	Planned withdrawal of life-sustaining therapy. Limiting and stopping treatment in the intensive care unit (ICU). Primary donor type (only type in some countries).
Controlled DCD IV	Circulatory arrest while brain dead	Circulatory arrest in a brain-dead candidate for donation.
Controlled DCD V	Medical assisted circulatory arrest	Expected circulatory arrest as a result of euthanasia (depend of countries legislation).

**Table 2:** Some of the most common preservation solutions and machines used in the clinic.

Preservation in Static Hypothermic Condition									
Organ Preservation Solutions Adaptable to Static or Sometimes Dynamic Preservation									
Solutions	K^+^ (mM)	Na^+^ (mM)	Buffer	pH	Impermeants	Adenosine (mM)	Anti-Oxidant	Colloid (g/L)	Organs
Blood	4.25	139	HCO^3−^	7.4	+	0	+	Albumin (50 g/L)	All
HTK (Custodiol^®^)	10	15	Histidine	7.2	+	5	-	-	Kidney, liver, pancreas, heart,
UW (Viaspan^®^) (Bridge to life^®^) (SPS-1^®^) (Bel-Gen^®^)	100	28.5	(K)H_2_PO_4_ HEPES	7.4	+	5	Glutathione	HES (50 g/L)	Kidney, liver, pancreas
Celsior^®^	15	100	HEPES	7.3	+	0	Glutathione	-	Kidney, liver, pancreas, heart, lung
IGL-1^®^	30	125	(K)H_2_PO_4_	7.3	+	5	Glutathione Allopurinol	PEG 35 kDa (1 g/L)	Kidney, liver, pancreas
SCOT 15^®^	5	118	HCO^3−^	7.4	+	0	-	PEG 20 kDa (15 g/L)	Kidney, liver
PERFADEX^®^ Plus	6	138	-	5.5	+	-	-	Dextran 40 (5%)	Lungs
**Preservation in dynamic condition**									
**Infusion machines**									
**Machine**		**Pulsatile perfusion**		**Temperature**			**Oxygenation**		
ORS—LifePort^®^				Hypothermia			No	Kidney	
IGL—WAVES^®^		+		Hypothermia			Yes	Kidney pancreas	
Kidney Assist (XVIVO)		+		Hypothermia to normothermia			Yes	Kidney	
Liver Assist (XVIVO)		+		Hypothermia to normothermia			Yes	Liver	
Lung Assist (XVIVO)		+/−		Hypothermia to normothermia			Yes	Lung	
VITASMART™ (Bridge to Life)				Hypothermia			Yes	Liver, kidney	
LIFECRADLE™ (Bridge to Life)				Hypothermia			Yes	Heart	
EVOSS™ (Bridge to Life)				Normothermia			Yes	Lung	
Organ Care System—OCS™ Lung (transmedics)		s+		Normothermia			Yes	Lung	
Organ Care System—OCS™ heart (transmedics)		+		Normothermia			Yes	Heart	
Organ Care System—OCS™ liver (transmedics)		+		Normothermia			Yes	Liver	
Steen Preservation Heart System (XVIVO)				Hypothermia				Heart	
XVIVO XPS™—XVIVO LS™ (XVIVO)				Normothermia			Yes	Lung	
Paragonix SherpaPak		+		Hypothermia			+/−	Heart, lung	
**Other solutions suitable for infusion machines**									
**Solutions**	**K^+^ (mM)**	**Na^+^ (mM)**	**Buffer**	**Impermeants**	**Anti-oxidant**		**Colloid (g/L)**	**Organs**	
KPS-1^®^	25	97.5	(K)H_2_PO_4_ HEPES	+	+		HES (50 g/L)	Kidney	
Belzer MPS^®^ PERF-GEN^®^	25	100	(K)H_2_PO_4_ HEPES	+	+		HES (50 g/L)	Kidney, liver, pancreas	
IGL2^®^	25	125	(K)H_2_PO_4_ Histidine	+	+		PEG 35 kDa (5g/L)	Liver, pancreas	
OCS Lung Solution	6	136	Phosphate	+			Dextran 40 (50 g/L)	Lung	
STEEN Solution ™	“low”		(Na)H_2_PO_4_	+			Human albumin Dextran 40	Lung	

## References

[1] ABM (2020). Rapport Médicale et Scientifique de l’agence de La Biomédecine. https://rams.agence-biomedecine.fr/.

[2] Smith M., Dominguez-Gil B., Greer D.M., Manara A.R., Souter M.J. (2019). Organ donation after circulatory death: Current status and future potential. Intensive Care Med..

[3] Port F.K., Bragg-Gresham J.L., Metzger R.A., Dykstra D.M., Gillespie B.W., Young E.W., Delmonico F.L., Wynn J.J., Merion R.M., Wolfe R.A. (2002). Donor characteristics associated with reduced graft survival: An approach to expanding the pool of kidney donors. Transplantation.

[4] Durand F., Antoine C., Soubrane O. (2019). Liver Transplantation in France. Liver Transplant..

[5] Bodzin A.S., Baker T.B. (2018). Liver Transplantation Today: Where We Are Now and Where We Are Going. Liver Transplant..

[6] Vanholder R., Domínguez-Gil B., Busic M., Cortez-Pinto H., Craig J.C., Jager K.J., Mahillo B., Stel V.S., Valentin M.O., Zoccali C. (2021). Organ donation and transplantation: A multi-stakeholder call to action. Nat. Rev. Nephrol..

[7] Resch T., Cardini B., Oberhuber R., Weissenbacher A., Dumfarth J., Krapf C., Boesmueller C., Oefner D., Grimm M., Schneeberger S. (2020). Transplanting Marginal Organs in the Era of Modern Machine Perfusion and Advanced Organ Monitoring. Front. Immunol..

[8] Hidalgo J.M.S., Rodríguez-Ortiz L., Arjona-Sánchez Á., Ayllón-Terán M., Gómez-Luque I., Ciria-Bru R., Luque-Molina A., López-Cillero P., Rufián-Peña S., Briceño-Delgado J. (2019). “Super-rapid” Technique in Donation After Circulatory Death Liver Donors: Advantages and Disadvantages. Transplant. Proc..

[9] Jochmans I., Hessheimer A.J., Neyrinck A.P., Paredes D., Bellini M.I., Dark J.H., Kimenai H.J.A.N., Pengel L.H.M., Watson C.J.E. (2021). ESOT Workstream 04 of the TLJ (Transplant Learning Journey) project Consensus Statement on Normothermic Regional Perfusion in Donation after Circulatory Death: Report from the European Society for Organ Transplantation’s Transplant Learning Journey. Transpl. Int..

[10] De Beule J., Jochmans I. (2020). Kidney Perfusion as an Organ Quality Assessment Tool—Are We Counting Our Chickens before They Have Hatched?. J. Clin. Med..

[11] Kerforne T., Allain G., Giraud S., Bon D., Ameteau V., Couturier P., Hebrard W., Danion J., Goujon J.-M., Thuillier R. (2019). Defining the optimal duration for normothermic regional perfusion in the kidney donor: A porcine preclinical study. Am. J. Transplant..

[12] Franzin R., Stasi A., Fiorentino M., Simone S., Oberbauer R., Castellano G., Gesualdo L. (2021). Renal Delivery of Pharmacologic Agents during Machine Perfusion to Prevent Ischaemia-Reperfusion Injury: From Murine Model to Clinical Trials. Front. Immunol..

[13] Arnoux V., Dorez D., Muller M., Gignoux A., Valignat C., Faucher A., Dechamboux E., Gour A.-S., Jakkel D., Saint Marcel L. (2014). Non-heart-beating renal donors: Organization in a non-university hospital. Prog. Urol..

[14] Carcy R., Cougnon M., Poet M., Durandy M., Sicard A., Counillon L., Blondeau N., Hauet T., Tauc M., Pisani D.F. (2021). Targeting oxidative stress, a crucial challenge in renal transplantation outcome. Free Radic. Biol. Med..

[15] Kerforne T., Favreau F., Thuillier R., Hauet T., Pinsard M. (2016). Toward a customized preservation for each kidney graft?. Nephrol. Ther..

[16] Fernández A.R., Sánchez-Tarjuelo R., Cravedi P., Ochando J., López-Hoyos M. (2020). Review: Ischemia Reperfusion Injury—A Translational Perspective in Organ Transplantation. Int. J. Mol. Sci..

[17] Dziodzio T., Biebl M., Pratschke J. (2014). Impact of brain death on ischemia/reperfusion injury in liver transplantation. Curr. Opin. Organ Transplant..

[18] Bon D., Chatauret N., Giraud S., Thuillier R., Favreau F., Hauet T. (2012). New strategies to optimize kidney recovery and preservation in transplantation. Nat. Rev. Nephrol..

[19] Eltzschig H.K., Bratton D.L., Colgan S.P. (2014). Targeting hypoxia signalling for the treatment of ischaemic and inflammatory diseases. Nat. Rev. Drug Discov..

[20] Kerforne T., Favreau F., Khalifeh T., Maiga S., Allain G., Thierry A., Dierick M., Baulier E., Steichen C., Hauet T. (2019). Hypercholesterolemia-induced increase in plasma oxidized LDL abrogated pro angiogenic response in kidney grafts. J. Transl. Med..

[21] Maïga S., Allain G., Hauet T., Roumy J., Baulier E., Scepi M., Dierick M., Van Hoorebeke L., Hannaert P., Guy F. (2017). Renal auto-transplantation promotes cortical microvascular network remodeling in a preclinical porcine model. PLoS ONE.

[22] Chatauret N., Favreau F., Giraud S., Thierry A., Rossard L., Le Pape S., Lerman L.O., Hauet T. (2014). Diet-induced increase in plasma oxidized LDL promotes early fibrosis in a renal porcine auto-transplantation model. J. Transl. Med..

[23] Barbier L., Ferhat M., Salamé E., Robin A., Herbelin A., Gombert J.-M., Silvain C., Barbarin A. (2019). Interleukin-1 Family Cytokines: Keystones in Liver Inflammatory Diseases. Front. Immunol..

[24] Issa N., Stephany B., Fatica R., Nurko S., Krishnamurthi V., Goldfarb D.A., Braun W.E., Dennis V.W., Heeger P.S., Poggio E.D. (2007). Donor Factors Influencing Graft Outcomes in Live Donor Kidney Transplantation. Transplantation.

[25] Hessheimer A.J., Billault C., Barrou B., Fondevila C. (2014). Hypothermic or normothermic abdominal regional perfusion in high-risk donors with extended warm ischemia times: Impact on outcomes?. Transpl. Int..

[26] Hosgood S.A., Brown R.J., Nicholson M.L. (2021). Advances in kidney preservation techniques and their application in clinical practice. Transplantation.

[27] Chazelas P., Steichen C., Favreau F., Trouillas P., Hannaert P., Thuillier R., Giraud S., Hauet T., Guillard J. (2021). Oxidative Stress Evaluation in Ischemia Reperfusion Models: Characteristics, Limits and Perspectives. Int. J. Mol. Sci..

[28] Steichen C., Giraud S., Bon D., Barrou B., Badet L., Salamé E., Kerforne T., Allain G., Roumy J., Jayle C. (2018). Barriers and Advances in Kidney Preservation. BioMed Res. Int..

[29] Southard M.J.H., Belzer M.F.O. (1995). ORGAN PRESERVATION. Annu. Rev. Med..

[30] Thuillier R., Giraud S., Favreau F., Goujon J.-M., Desurmont T., Eugene M., Barrou B., Hauet T. (2011). Improving Long-Term Outcome in Allograft Transplantation: Role of Ionic Composition and Polyethylene Glycol. Transplantation.

[31] Giraud S., Codas R., Hauet T., Eugene M., Badet L. (2014). Polyethylene glycols and organ protection against I/R injury. Prog. Urol..

[32] Chen Y., Shi J., Xia T.C., Xu R., He X., Xia Y. (2019). Preservation Solutions for Kidney Transplantation: History, Advances and Mechanisms. Cell Transplant..

[33] Petrenko A., Carnevale M., Somov A., Osorio J., Rodríguez J., Guibert E., Fuller B., Froghi F. (2019). Organ Preservation into the 2020s: The Era of Dynamic Intervention. Transfus. Med. Hemother..

[34] Chatauret N., Coudroy R., Delpech P.O., Vandebrouck C., Hosni S., Scepi M., Hauet T. (2014). Mechanistic Analysis of Nonoxygenated Hypothermic Machine Perfusion’s Protection on Warm Ischemic Kidney Uncovers Greater eNOS Phosphorylation and Vasodilation. Am. J. Transplant..

[35] Brat A., de Vries K.M., van Heurn E.W., Huurman V.A., de Jongh W., Leuvenink H.G., van Zuilen A.D., Haase-Kromwijk B.J., de Jonge J., Berger S.P. (2021). Hypothermic Machine Perfusion as a National Standard Preservation Method for Deceased Donor Kidneys. Transplantation.

[36] Singh N., Logan A., Schenk A., Bumgardner G., Brock G., El-Hinnawi A., Rajab A., Washburn K. (2021). Machine perfusion of kidney allografts affects early but not late graft function. Am. J. Surg..

[37] Val A.R., Guarrera J., Porte R.J., Selzner M., Spiro M., Raptis D.A., Friend P.J. (2022). Does machine perfusion improve immediate and short-term outcomes by enhancing graft function and recipient recovery after liver transplantation?—A systematic review of the literature, meta-analysis and expert panel recommendations. Clin. Transplant..

[38] Qin G., Jernryd V., Sjöberg T., Steen S., Nilsson J. (2022). Machine Perfusion for Human Heart Preservation: A Systematic Review. Transpl. Int..

[39] Dirito J.R., Hosgood S.A., Tietjen G.T., Nicholson M.L. (2018). The future of marginal kidney repair in the context of normothermic machine perfusion. Am. J. Transplant..

[40] Elliott T.R., Nicholson M.L., Hosgood S.A. (2021). Normothermic kidney perfusion: An overview of protocols and strategies. Am. J. Transplant..

[41] Hamar M., Urbanellis P., Kaths M.J., Kollmann D., Linares I., Ganesh S., Wiebe A., Cen J.Y., Yip P.M., John R. (2018). Normothermic Ex Vivo Kidney Perfusion Reduces Warm Ischemic Injury of Porcine Kidney Grafts Retrieved after Circulatory Death. Transplantation.

[42] Pool M.B.F., Hamelink T.L., van Goor H., Heuvel M.C.V.D., Leuvenink H.G.D., Moers C. (2021). Prolonged ex-vivo normothermic kidney perfusion: The impact of perfusate composition. PLoS ONE.

[43] Hosgood S.A., Nicholson M.L. (2021). A Short Period of Normothermic Machine Perfusion May Not Be Able to Predict Primary Nonfunction in Uncontrolled Circulatory Death Kidneys. Transplantation.

[44] Hosgood S., Saeb-Parsy K., Wilson C., Callaghan C., Collett D., Nicholson M.L. (2017). Protocol of a randomised controlled, open-label trial of ex vivo normothermic perfusion versus static cold storage in donation after circulatory death renal transplantation. BMJ Open.

[45] Rijkse E., Bouari S., Kimenai H.J.A.N., de Jonge J., de Bruin R.W.F., Slagter J.S., Hoogen M.W.F.V.D., Ijzermans J.N.M., Hoogduijn M.J., Minnee R.C. (2021). Additional Normothermic Machine Perfusion versus Hypothermic Machine Perfusion in Suboptimal Donor Kidney Transplantation: Protocol of a Randomized, Controlled, Open-Label Trial. Int. J. Surg. Protoc..

[46] Muller X., Mohkam K., Mueller M., Schlegel A., Dondero F., Sepulveda A., Savier E., Scatton O., Bucur P., Salame E. (2020). Hypothermic Oxygenated Perfusion Versus Normothermic Regional Perfusion in Liver Transplantation From Controlled Donation after Circulatory Death: First International Comparative Study. Ann. Surg..

[47] Hessheimer A.J., Riquelme F., Fundora-Suárez Y., Pérez R.G., Fondevila C. (2019). Normothermic perfusion and outcomes after liver transplantation. Transplant. Rev..

[48] Nasralla D., Coussios C.C., Mergental H., Akhtar M.Z., Butler A.J., Ceresa C.D.L., Chiocchia V., Dutton S.J., García-Valdecasas J.C., Heaton N. (2018). A randomized trial of normothermic preservation in liver transplantation. Nature.

[49] Cypel M., Yeung J., Liu M., Anraku M., Chen F., Karolak W., Sato M., Laratta J., Azad S., Madonik M. (2011). Normothermic Ex Vivo Lung Perfusion in Clinical Lung Transplantation. N. Engl. J. Med..

[50] Loor G., Warnecke G., Villavicencio M.A., Smith M.A., Kukreja J., Ardehali A., Hartwig M., Daneshmand M.A., Hertz M.I., Huddleston S. (2019). Portable normothermic ex-vivo lung perfusion, ventilation, and functional assessment with the Organ Care System on donor lung use for transplantation from extended-criteria donors (EXPAND): A single-arm, pivotal trial. Lancet Respir. Med..

[51] Niederberger P., Farine E., Raillard M., Dornbierer M., Freed D.H., Large S.R., Chew H.C., MacDonald P.S., Messer S.J., White C.W. (2019). Heart Transplantation with Donation after Circulatory Death. Circ. Heart Fail..

[52] Minor T., von Horn C. (2020). Reduction of Renal Preservation/Reperfusion Injury by Controlled Hyperthermia During Ex Vivo Machine Perfusion. Clin. Transl. Sci..

[53] Fabry G., Doorschodt B.M., Grzanna T., Boor P., Elliott A., Stollenwerk A., Tolba R.H., Rossaint R., Bleilevens C. (2019). Cold Preflush of Porcine Kidney Grafts Prior to Normothermic Machine Perfusion Aggravates Ischemia Reperfusion Injury. Sci. Rep..

[54] Kaths J.M., Echeverri J., Chun Y.M., Cen J.Y., Goldaracena N., Linares I., Dingwell L., Yip P., John R., Bagli D. (2017). Continuous Normothermic Ex Vivo Kidney Perfusion Improves Graft Function in Donation after Circulatory Death Pig Kidney Transplantation. Transplantation.

[55] Weissenbacher A., Faro M.L.L., Boubriak O., Soares M.F., Roberts I.S., Hunter J.P., Voyce D., Mikov N., Cook A., Ploeg R.J. (2019). Twenty-four–hour normothermic perfusion of discarded human kidneys with urine recirculation. Am. J. Transplant..

[56] Minor T., Von Horn C., Gallinat A., Kaths M., Kribben A., Treckmann J., Paul A. (2020). First-in-man controlled rewarming and normothermic perfusion with cell-free solution of a kidney prior to transplantation. Am. J. Transplant..

[57] Jochmans I., Nicholson M.L., Hosgood S.A. (2017). Kidney perfusion: Some like it Hot Others Prefer to Keep it Cool. Curr. Opin. Organ Transplant..

[58] Hamelink T.L., Ogurlu B., De Beule J., Lantinga V.A., Pool M.B., Venema L.H., Leuvenink H.G., Jochmans I., Moers C. (2021). Renal Normothermic Machine Perfusion: The Road Toward Clinical Implementation of a Promising Pretransplant Organ Assessment Tool. Transplantation.

[59] Xu J., Buchwald J., Martins P.N. (2020). Review of Current Machine Perfusion Therapeutics for Organ Preservation. Transplantation.

[60] Minor T., von Horn C., Paul A. (2017). Role of temperature in reconditioning and evaluation of cold preserved kidney and liver grafts. Curr. Opin. Organ Transplant..

[61] Kasil A., Giraud S., Couturier P., Amiri A., Danion J., Donatini G., Matillon X., Hauet T., Badet L. (2019). Individual and Combined Impact of Oxygen and Oxygen Transporter Supplementation during Kidney Machine Preservation in a Porcine Preclinical Kidney Transplantation Model. Int. J. Mol. Sci..

[62] Jochmans I., Brat A., Davies L., Hofker H.S., van de Leemkolk F.E.M., Leuvenink H.G.D., Knight S.R., Pirenne J., Ploeg R.J., COMPARE Trial Collaboration and Consortium for Organ Preservation in Europe (COPE) (2020). Oxygenated versus standard cold perfusion preservation in kidney transplantation (COMPARE): A randomised, double-blind, paired, phase 3 trial. Lancet.

[63] Peters S.M., Rauen U., Tijsen M.J., Bindels R.J., van Os C.H., de Groot H., Wetzels J.F. (1998). Cold preservation of isolated rabbit proximal tubules induces radical-mediated cell Injury. Transplantation.

[64] Patel K., Smith T.B., Neil D.A., Thakker A., Tsuchiya Y., Higgs E.B., Hodges N.J., Ready A.R., Nath J., Ludwig C. (2019). The Effects of Oxygenation on Ex Vivo Kidneys Undergoing Hypothermic Machine Perfusion. Transplantation.

[65] Hoyer D.P., Gallinat A., Swoboda S., Wohlschlaeger J., Rauen U., Paul A., Minor T. (2014). Influence of Oxygen Concentration during Hypothermic Machine Perfusion on Porcine Kidneys from Donation after Circulatory Death. Transplantation.

[66] Darius T., Gianello P., Vergauwen M., Mourad N., Buemi A., De Meyer M., Mourad M. (2018). The effect on early renal function of various dynamic preservation strategies in a preclinical pig ischemia-reperfusion autotransplant model. Am. J. Transplant..

[67] Thuillier R., Allain G., Celhay O., Hebrard W., Barrou B., Badet L., Leuvenink H., Hauet T. (2013). Benefits of active oxygenation during hypothermic machine perfusion of kidneys in a preclinical model of deceased after cardiac death donors. J. Surg. Res..

[68] Meister F.A., Czigany Z., Rietzler K., Miller H., Reichelt S., Liu W.-J., Boecker J., Moeller M.J., Tolba R.H., Hamesch K. (2020). Decrease of renal resistance during hypothermic oxygenated machine perfusion is associated with early allograft function in extended criteria donation kidney transplantation. Sci. Rep..

[69] Husen P., Boffa C., Jochmans I., Krikke C., Davies L., Mazilescu L., Brat A., Knight S., Wettstein D., Cseprekal O. (2021). Oxygenated End-Hypothermic Machine Perfusion in Expanded Criteria Donor Kidney Transplant; A Randomized Clinical Trial. JAMA Surg..

[70] Mazilescu L.I., Urbanellis P., Kaths M.J., Ganesh S., Goto T., Noguchi Y., John R., Konvalinka A., Mucsi I., Ghanekar A. (2021). Prolonged Normothermic Ex Vivo Kidney Perfusion Is Superior to Cold Nonoxygenated and Oxygenated Machine Perfusion for the Preservation of DCD Porcine Kidney Grafts. Transplant. Direct.

[71] Thuillier R., Delpy E., Matillon X., Kaminski J., Kasil A., Soussi D., Danion J., Sauvageon Y., Rod X., Donatini G. (2019). Preventing acute kidney injury during transplantation: The application of novel oxygen carriers. Expert Opin. Investig. Drugs.

[72] Bhattacharjee R.N., Patel S.V., Sun Q., Jiang L., Richard-Mohamed M., Ruthirakanthan A., Aquil S., Al-Ogaili R., Juriasingani S., Sener A. (2020). Renal Protection Against Ischemia Reperfusion Injury: Hemoglobin-based Oxygen Carrier-201 Versus Blood as an Oxygen Carrier in Ex Vivo Subnormothermic Machine Perfusion. Transplantation.

[73] Alix P., Val-Laillet D., Turlin B., Ben Mosbah I., Burel A., Bobillier E., Bendavid C., Delpy E., Zal F., Corlu A. (2020). Adding the oxygen carrier M101 to a cold-storage solution could be an alternative to HOPE for liver graft preservation. JHEP Rep..

[74] Sakai H., Tomiyama K.-I., Sou K., Takeoka S., Tsuchida E. (2000). Poly(ethylene glycol)-Conjugation and Deoxygenation Enable Long-Term Preservation of Hemoglobin-Vesicles as Oxygen Carriers in a Liquid State. Bioconj. Chem..

[75] Mot A.C., Roman A., Lupan I., Kurtz D.M., Silaghi-Dumitrescu R. (2010). Towards the Development of Hemerythrin-Based Blood Substitutes. J. Protein Chem..

[76] Mallet V., Dutheil D., Polard V., Rousselot M., Leize E., Hauet T., Goujon J.M., Zal F. (2014). Dose-Ranging Study of the Performance of the Natural Oxygen Transporter HEMO2Life in Organ Preservation. Artif. Organs.

[77] Le Meur Y., Badet L., Essig M., Thierry A., Büchler M., Drouin S., Deruelle C., Morelon E., Pesteil F., Delpech P. (2020). First-in-human use of a marine oxygen carrier (M101) for organ preservation: A safety and proof-of-principle study. Am. J. Transplant..

[78] van Leeuwen O., De Vries Y., Fujiyoshi M., Nijsten M.W.N., Ubbink R., Pelgrim G.J., Werner M.J.M., Reyntjens K.M.E.M., Berg A.P.V.D., De Boer M.T. (2019). Transplantation of High-risk Donor Livers After Ex Situ Resuscitation and Assessment Using Combined Hypo- and Normothermic Machine Perfusion: A Prospective Clinical Trial. Ann. Surg..

[79] De Vries Y., Matton A.P.M., Nijsten M.W.N., Werner M.J.M., van den Berg A.P., De Boer M.T., Buis C.I., Fujiyoshi M., De Kleine R.H.J., van Leeuwen O. (2019). Pretransplant sequential hypo- and normothermic machine perfusion of suboptimal livers donated after circulatory death using a hemoglobin-based oxygen carrier perfusion solution. Am. J. Transplant..

[80] He X., Guo Z., Zhao Q., Ju W., Wang D., Wu L., Yang L., Ji F., Tang Y., Zhang Z. (2018). The first case of ischemia-free organ transplantation in humans: A proof of concept. Am. J. Transplant..

[81] He X., Chen G., Zhu Z., Zhang Z., Yuan X., Han M., Zhao Q., Zheng Y., Tang Y., Huang S. (2019). The First Case of Ischemia-Free Kidney Transplantation in Humans. Front. Med..

[82] Mesnard B., Ogbemudia A.E., Karam G., Dengu F., Hackim G., Rigaud J., Blancho G., Drouin S., Timsit M.O., Branchereau J. (2021). What is the evidence for oxygenation during kidney preservation for transplantation in 2021? A scoping review. World J. Urol..

[83] De Vries R.J., Tessier S.N., Banik P.D., Nagpal S., Cronin S.E.J., Ozer S., Hafiz E.O.A., Van Gulik T.M., Yarmush M.L., Markmann J.F. (2019). Supercooling extends preservation time of human livers. Nat. Biotechnol..

[84] De Vries R.J., Tessier S.N., Banik P.D., Nagpal S., Cronin S.E.J., Ozer S., Hafiz E.O.A., Van Gulik T.M., Yarmush M.L., Markmann J.F. (2020). Subzero non-frozen preservation of human livers in the supercooled state. Nat. Protoc..

[85] Chandak P., Phillips B., Uwechue R., Thompson E., Bates L., Ibrahim I., Sewpaul A., Figueiredo R., Olsburgh J., Hosgood S. (2019). Dissemination of a novel organ perfusion technique: Ex vivo normothermic perfusion of deceased donor kidneys. Artif. Organs.

[86] Bruinsma B.G., Uygun K. (2017). Subzero organ preservation: The Dawn of a New Ice Age?. Curr. Opin. Organ Transplant..

[87] Buchwald J., Xu J., Bozorgzadeh A., Martins P.N. (2020). Therapeutics administered during ex vivo liver machine perfusion: An overview. World J. Transplant..

[88] Melis N., Rubera I., Cougnon M., Giraud S., Mograbi B., Belaid A., Pisani D., Huber S.M., Lacas-Gervais S., Fragaki K. (2017). Targeting eIF5A Hypusination Prevents Anoxic Cell Death through Mitochondrial Silencing and Improves Kidney Transplant Outcome. J. Am. Soc. Nephrol..

[89] Giraud S., Kerforne T., Zely J., Ameteau V., Couturier P., Tauc M., Hauet T. (2020). The inhibition of eIF5A hypusination by GC7, a preconditioning protocol to prevent brain death-induced renal injuries in a preclinical porcine kidney transplantation model. Am. J. Transplant..

[90] Barrera-Chimal J., Jaisser F. (2020). Pathophysiologic mechanisms in diabetic kidney disease: A focus on current and future therapeutic targets. Diabetes Obes. Metab..

[91] Barrera-Chimal J., André-Grégoire G., Cat A.N.D., Lechner S.M., Cau J., Prince S., Kolkhof P., Loirand G., Sauzeau V., Hauet T. (2017). Benefit of Mineralocorticoid Receptor Antagonism in AKI: Role of Vascular Smooth Muscle Rac1. J. Am. Soc. Nephrol..

[92] Rao P.S., Schaubel D.E., Guidinger M.K., Andreoni K.A., Wolfe R.A., Merion R.M., Port F.K., Sung R.S. (2009). A Comprehensive Risk Quantification Score for Deceased Donor Kidneys: The Kidney Donor Risk Index. Transplantation.

[93] Querard A.-H., Foucher Y., Combescure C., Dantan E., Larmet D., Lorent M., Pouteau L.-M., Giral M., Gillaizeau F. (2016). Comparison of survival outcomes between Expanded Criteria Donor and Standard Criteria Donor kidney transplant recipients: A systematic review and meta-analysis. Transpl. Int..

[94] Loupy A., Aubert O., Orandi B.J., Naesens M., Bouatou Y., Raynaud M., Divard G., Jackson A.M., Viglietti D., Giral M. (2019). Prediction system for risk of allograft loss in patients receiving kidney transplants: International derivation and validation study. BMJ.

[95] Benmoussa K., Garaude J., Acín-Pérez R. (2018). How Mitochondrial Metabolism Contributes to Macrophage Phenotype and Functions. J. Mol. Biol..

[96] Bekkering S., Domínguez-Andrés J., Joosten L.A., Riksen N.P., Netea M.G. (2021). Trained Immunity: Reprogramming Innate Immunity in Health and Disease. Annu. Rev. Immunol..

[97] Domínguez-Andrés J., Fanucchi S., Joosten L.A., Mhlanga M.M., Netea M.G. (2020). Advances in understanding molecular regulation of innate immune memory. Curr. Opin. Cell Biol..

